# ADULT DAY SERVICES IN A RURAL REGION: CHALLENGES AND OPPORTUNITIES

**DOI:** 10.1093/geroni/igz038.555

**Published:** 2019-11-08

**Authors:** Sandra S Butler, Jennifer Crittenden, Dyan Walsh, Lenard Kaye

**Affiliations:** 1 University of Maine, Orono, Maine, United States; 2 UMaine Center on Aging, University of Maine, Bangor, Maine, United States; 3 Eastern Area Agency on Aging, Bangor, Maine, United States

## Abstract

Adult day services (ADS) programs provide stimulation and socialization for older adults with cognitive and physical disabilities, and much needed respite for family caregivers. Like many services for older adults, ADS programs are far less available in rural regions of the country than in more urban settings. This paper reports on a needs assessment for an ADS program in a small city, which serves as a health and human services hub for a large rural area; a particular focus of the study was to assess the feasibility and interest in intergenerational programming. Family caregivers were surveyed (n = 84) about their use and knowledge of and interest in ADS. Less than one in five respondents were using or had ever used ADS. Cost (20%) and ignorance of such programs (20%) were primary reasons for not using ADS; reduction of stress was the most frequently cited reason for using ADS (73%). Ten in-person interviews were conducted with ADS program directors and service providers who refer clients to ADS. Funding issues emerged as the key challenge given lack of private insurance coverage and poor reimbursement levels from public insurance programs. Challenges around transportation, stigma, and marketing of services also surfaced in the interviews. Nonetheless, all ten informants spoke of the positive impact of ADS for both consumers and their caregivers, and generally endorsed intergenerational activities, though with caveats. Implications will be discussed, including the need for greater financial support for this valuable aspect of our long-term supports and services system.

